# Outcomes associated with large loop excision of the cervical transformation zone in women 60–64 years of age: A population‐based register study from Denmark

**DOI:** 10.1111/aogs.15111

**Published:** 2025-04-07

**Authors:** Julie Laub Erdal, Reza Rafiolsadat Serizawa, Matejka Rebolj, Jeppe Bennekou Schroll

**Affiliations:** ^1^ Department of Clinical Medicine University of Copenhagen Copenhagen Denmark; ^2^ Department of Pathology Copenhagen University Hospital Amager and Hvidovre Hvidovre Denmark; ^3^ Centre for Cancer Screening, Prevention and Early Diagnosis, Wolfson Institute of Population Health Queen Mary University of London London UK; ^4^ Department of Clinical Research, Centre for Evidence‐Based Medicine Odense and Cochrane Denmark University of Southern Denmark Odense Denmark; ^5^ Open Patient Data Explorative Network Odense University Hospital Odense Denmark; ^6^ Department of Gynecology & Obstetrics Herlev‐Gentofte Hospital Hellerup Denmark

**Keywords:** cervical cancer, cervical intraepithelial neoplasia, cytology, human papillomavirus, loop electrosurgical excision procedure, screening

## Abstract

**Introduction:**

For women treated for cervical dysplasia at 60–64 years in Denmark, we reported the frequency of abnormalities before and after treatment of cervix uteri (most frequently performed as large loop excision of the cervical transformation zone, LLETZ) using population‐based real‐world data.

**Material and Methods:**

We conducted a retrospective cohort study based on national data from the Danish Pathology Data Bank and identified women who underwent a LLETZ in 2010–2016 at the age of 60–64. Women were managed according to nationwide evidence‐based recommendations proposed by the Danish professional organizations. We retrieved information on all LLETZ specimens, cervical histology, cytology, and human papillomavirus (HPV) tests in the period of 2 years prior to the procedure to 2 years thereafter. We reported the frequencies of abnormalities before, at, or after the procedure.

**Results:**

Of the 1014 women who had a LLETZ during the study period, 660 (65%) showed cervical intraepithelial neoplasia grade 1 or worse (CIN1+, including CIN1, CIN2, CIN3, cervical cancer, and CIN not otherwise specified) in their LLETZ specimen, with free resection margins in 255 (39%). Of the 1014 women, 551 (54%) had CIN2+ in a biopsy preceding the LLETZ and in 567 (56%) CIN2+ was found in their LLETZ specimen. In 37 (4%) women, the specimen showed cervical cancer; whereas in the pre‐LLETZ biopsies of these 37 women, cancer was detected in only 7 (1%). After LLETZ, 818 (81%) women underwent test‐of‐cure follow‐up which was positive in 406 women (40%). Furthermore, 408 (40%) women had new histological samples registered after LLETZ. These showed CIN2+ in 134 (13%) women, whereas a new cancer was diagnosed in 11 (1%) women.

**Conclusions:**

Due to persistent abnormal tests after LLETZ, an extended follow‐up is still required for a large proportion of the women in this age group.

## Graphical abstract

Surgical removal of cervical tissue at age 60–64 identifies occult lesions, but does not eradicate HPV infections in almost half of the women. This information can help inform women and doctors before undertaking such procedures.

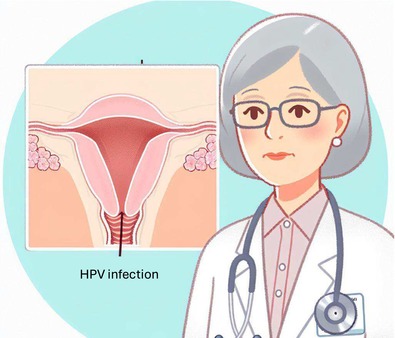

**Abbreviations**
AIS: adenocarcinoma in situ
ASC‐H: atypical squamous cells, high‐grade squamous intraepithelial lesions cannot be excluded.
ASCUS: atypical squamous cells of undetermined significance
CIN: cervical intraepithelial neoplasia
HPV: human papillomavirus
LLETZ: large loop excision of the cervical transformation zone
LSIL: low‐grade squamous intraepithelial lesion
NOS: not otherwise specified

**Key message**

Surgical removal of cervical tissue at age 60–64 identifies occult lesions, but does not eradicate HPV infections in almost half of the women.

## 1 INTRODUCTION

The increasing use of human papillomavirus (HPV) testing for primary cervical cancer screening is posing several new challenges for the management of the affected women. Some of those revolve around the prognosis of unresolved HPV infections and other abnormalities in women nearing the upper age of their eligibility for screening. Furthermore, it is challenging for the clinician to rule out disease located in the cervical canal. At the age of 60 and over, when most women show signs of post‐menopausal anatomical changes of the cervix, around 5% still test positive for HPV.^1^ Although at this age more than 80% of HPV‐positive women have no high‐grade cervical intraepithelial neoplasia or worse (CIN2+) that could be resolved by treatment, many are unable to clear their infections.^1^, ^2^

Persistent HPV infections are a cause for concern in terms of interpreting women's residual risk of cervical cancer because continued screening beyond the age of 65 years is not recommended in several countries.^3^, ^4^, ^5^ As surgical treatment is usually not recommended in these women, they may end up in an extended follow‐up with additional tests and colposcopies that can span several years.^6^ The same problem applies to women who undergo a large loop excision of the cervical transformation zone (LLETZ) for biopsy‐confirmed lesions and who are still HPV‐positive after the procedure. In Denmark, testing for HPV together with cytology 6 months after LLETZ^7^, ^8^ has been used as a test of cure since 2012, although in parts of the country this protocol had been implemented even earlier. Owing to a lack of long‐term observational data, however, specifying clear evidence‐based conditions under which older women could be ceased from further follow‐up is associated with considerable difficulties. In practice, management decisions tend to be left to clinicians. To expedite the process in women with less severe abnormalities or in case of persistent HPV infections, clinicians sometimes opt for surgical excision of the cervical tissue, typically as a LLETZ. In Denmark, for example, this pragmatic approach is based on a consensus between clinicians and patients, as women find LLETZ acceptable if it could shorten the follow‐up.^9^

The primary aim of this population‐based study using registry data from Denmark was to understand the value of a LLETZ procedure for a correct diagnosis in women aged 60–64 years, and to provide information on the likely outcomes after the procedure. We do this by reporting the cytology, HPV testing, and histology outcomes leading up to a LLETZ procedure and those from samples taken after the LLETZ. A secondary aim was to report the outcomes for the subgroup of women who only had a positive HPV test prior to the LLETZ procedure, as they are a particularly challenging group to manage.

## 2 MATERIAL AND METHODS

In Denmark, women aged 50–64 years are invited for screening if they have no test registered in the previous 5 years. In 2012, HPV testing started replacing cytology as the primary screening method for women aged 60–64 years. Women aged 60–64 years with a negative HPV test and no unresolved cervical abnormalities can exit the screening programme and will not receive additional invitations. The last of the five Danish regions switched to HPV‐based screening in this age group in August 2014.^1^ At present, women are directly referred to colposcopy if their cervical samples show an HPV infection with genotypes HPV16/18 irrespective of cytology, or if they are infected with any of the other 12 high‐risk HPV genotypes combined with atypical squamous cells of undetermined significance or worse (≥ASCUS). Other HPV‐positive women are recommended for an early recall in 12 months. Genotyping was, however, not used consistently in the early period.^1^

In Denmark, it is recommended to take directed cervical biopsies, and, in case of apparently negative colposcopy findings, at least four random biopsies. All women with CIN2+ are recommended to undergo treatment, which is usually performed as LLETZ. However, the guidelines do not specify the recommended follow‐up of women with persistently HPV‐positive tests and normal or inadequate cytology and/or histology. Six months after LLETZ, cytology and HPV testing is recommended. In the case of involved or indeterminate margins at LLETZ, an additional HPV and cytology test in 12 months is recommended. Either abnormal cytology or a positive HPV test would prompt a referral to colposcopy. No genotyping is used at this stage.

This retrospective cohort study is based on data that included all cervical samples registered in the Danish Pathology Data Bank (Patobank)^10^ between January 1, 2008 and December 31, 2018 from women residing in Denmark and born between 1944 and 1956. The Patobank is a nationwide real‐time electronic register of pathological tissue and cell sample examinations, collected in hospitals or by private specialists, and has been considered virtually complete since the mid‐2000s.^11^ Topography and morphology of the sample material are classified according to the Danish version of the Systematized Nomenclature of Medicine (SNOMED). Records belonging to the same woman were linked using the Danish unique personal identification numbers, so women could be traced even when they moved to a different part of the country.

We included women aged 60–64 years at the time of the “index” LLETZ between 2010 and 2016 (for brevity, the “index” LLETZ is referred to as “LLETZ” in the remainder of the manuscript). All LLETZ were identified in the Patobank with SNOMED codes T83701 or T83702, which also include needle excision. We restricted our study group to LLETZs undertaken while HPV testing was part of the standard test‐of‐cure management. In Denmark, laboratories operate within defined, non‐overlapping geographical areas. To establish the start date of HPV test‐of‐cure testing at each laboratory, we conducted an analysis of the annual frequency of test types used for post‐LLETZ management. This resulted in the inclusion of LLETZs from 2010 to 2013 at the earliest, depending on the laboratory. Furthermore, to ensure that all women included in the study had a minimum of 2 years of follow‐up data, we concluded the inclusion period on December 31, 2016.

For all LLETZs, we determined their histological outcomes, material (in)sufficiency, and margin status (free, involved/indeterminate, not registered). Histological outcomes were classified as CIN (Grades 1, 2, 3, not otherwise specified [NOS], or adenocarcinoma in situ [AIS]) or cervical cancer. In line with the routine practice in Denmark, the category of CIN2+ included cases with CIN NOS, CIN2, CIN3, AIS, and cervical cancer, whereas the category of CIN3+ included CIN3, AIS, and cervical cancer. Where the outcomes are reported for the group of women with CIN1+, these include CIN1 and CIN2+ as defined above. Samples not including the transformation zone (a Patobank record with a SNOMED code indicating that the transformation zone is not represented) or with material unsuitable for diagnostic assessment for other reasons (SNOMED codes indicating lost, too little, scarce, unrepresentative, or no material) were considered insufficient. When both the endocervical and ectocervical margin status was reported, the margins were considered insufficient if either of the two margins was considered insufficient. Cytology was classified according to the Bethesda classification and categorized as negative, abnormal (≥ASCUS), or insufficient (the latter if registered with a SNOMED code indicating that the material was unsuitable for diagnosis). HPV test outcomes were reported as positive or negative for DNA from the 14 high‐risk HPV genotypes. Tests registered within 6 days of the LLETZ specimen were excluded, as they were likely taken at the same time but registered on separate dates.

We reported the characteristics of the LLETZ specimens in the whole study group in Table 1. Histological outcomes up to 2 years before the LLETZ procedure were described for the whole study group in Table 2. These pre‐LLETZ outcomes, as well as those reported in 2 years after the LLETZ procedure, were shown in more detail separately for women with CIN2+, CIN3+, or cervical cancer in the LLETZ specimen in Table 3. Post‐LLETZ histological outcomes are given for the whole study group in Table 4; this table also includes the results of the test‐of‐cure cytology and HPV tests. Finally, we identified the group of women whose only indication for LLETZ appeared to be an HPV‐positive test without abnormal cytology or histology. Table 5 reports the histological findings in their LLETZ specimens as well as their test‐of‐cure test results.

**TABLE 1:** The study population of 1014 women who had a LLETZ procedure at age 60–64 years.

	*N* (%)
Total	1014 (100%)
Age (years), median (IQR)	62 (61–63)
Calendar year	
2010–2013	302 (30%)
2014–2016	712 (70%)
Histological findings in the specimen	
Insufficient	72 (7%)
No CIN or CIN1	375 (37%)
CIN2+	567 (56%)
CIN3+	355 (35%)
Cervical cancer	37 (4%)
Margin status among women with CIN1+ (*n* = 660, 100%)	
Free	255 (39%)
Involved or undetermined	361 (55%)
Resection margin not described	44 (7%)

*Note*: CIN2+ includes CIN2 and CIN3+, and CIN3+ includes CIN3 and cervical cancer. Both squamous and glandular lesions are included in these counts.

Abbreviations: CIN, cervical intraepithelial neoplasia; IQR, interquartile range; LLETZ, large loop excision of the transformation zone.

**TABLE 2:** Worst histological findings up to 24 months before the LLETZ treatment (*N* = 1014).

Worst histological finding in the preceding 24 months	*N* (%)
Any biopsy	869 (86%)
Insufficient	109 (11%)
No CIN or CIN1	209 (21%)
CIN2+	551 (54%)
CIN3+	250 (25%)
Cervical cancer	13 (1%)
No biopsies	145 (14%)

*Note*: CIN2+ includes CIN2 and CIN3+, and CIN3+ includes CIN3 and cervical cancer. Both squamous and glandular lesions are included in these counts.

Abbreviations: CIN, cervical intraepithelial neoplasia; LLETZ, large loop excision of the transformation zone.

**TABLE 3:** Women with CIN2+, CIN3+, or cervical cancer in the LLETZ specimen: The worst histological findings in 24 months before and after LLETZ.

	Worst histology in 24 months pre‐LLETZ	Worst histology in 24 months post‐LLETZ
CIN2+ in the LLETZ specimen	567 (100%)	567 (100%)
No histology	40 (7%)	319 (56%)
<CIN2+^a^	120 (21%)	145 (26%)
CIN2+	407 (72%)	103 (18%)
Cervical cancer	9 (2%)	26^b^ (5%)
CIN3+ in the LLETZ specimen	432 (100%)	432 (100%)
None or <CIN2^a^	111 (26%)	340 (79%)
CIN2+	321 (74%)	92 (21%)
Cervical cancer	9 (2%)	25^b^ (6%)
Cancer in the LLETZ specimen	37 (100%)	37 (100%)
None or <CIN2^a^	9 (24%)	NR
CIN2+	28 (76%)	NR
Cervical cancer	7 (19%)	NR

*Note*: CIN2+ includes CIN2, CIN3, and cervical cancer. Both squamous and glandular lesions are included in these counts.

Abbreviations: CIN, cervical intraepithelial neoplasia; LLETZ, large loop excision of the transformation zone; NR, not relevant.

^a^ Includes insufficient samples.

^b^ Includes women in whom cancer was already present in the LLETZ sample.

**TABLE 4:** Histological, HPV testing, and cytological findings after LLETZ.

	*N* (%)
Total	1014 (100%)
The first ToC test	
Days from LLETZ to the first ToC test (median, IQR)	194 (169–226)
None	145 (14%)
HPV tests	822 (81%)
Cytology tests	865 (85%)
Both HPV and cytology tests	818 (81%)
Positive ToC^a^	406 (40%)
Negative ToC^b^	412 (41%)
Outcome of the worst histology test in 24 months	
Any biopsy	408 (40%)
Insufficient	73 (7%)
<CIN2	201 (20%)
CIN2+	134 (13%)
Cervical cancer^c^	30 (3%)
No biopsies	606 (60%)

*Note*: CIN2+ includes CIN2, CIN3, and cervical cancer. Both squamous and glandular lesions are included in these counts.

Abbreviations: CIN, cervical intraepithelial neoplasia; HPV, human papillomavirus; IQR, interquartile range; LLETZ, large loop excision of the transformation zone; ToC, test of cure, that is, the recommended HPV and cytology follow‐up testing after LLETZ treatment.

^a^ Defined as HPV‐positive or atypical squamous cells of undetermined significance or worse.

^b^ Defined as HPV‐negative and normal cytology.

^c^ Of which 19 were cases that were also diagnosed in the LLETZ specimen, and 11 were new cases.

**TABLE 5:** The 108 women whose prior test before the LLETZ procedure was a positive HPV test: Findings in the LLETZ specimen and the first test‐of‐cure results following LLETZ.

	*N* (%)
Total	108 (100%)
Histological findings in the LLETZ specimen	
CIN2+	18 (17%)
CIN3+	12 (11%)
The first ToC test	
Days from LLETZ to the first ToC test (median, IQR)	215 (183–264)
None	13 (12%)
HPV tests	95 (88%)
Cytology tests	95 (88%)
Both HPV and cytology tests	95 (88%)
Positive ToC^a^	53 (49%)
Negative ToC^b^	42 (39%)

*Note*: CIN2+ includes CIN2 and CIN3+, and CIN3+ includes CIN3 and cervical cancer. Both squamous and glandular lesions are included in these counts.

Abbreviations: CIN, cervical intraepithelial neoplasia; HPV, human papillomavirus; IQR, interquartile range; LLETZ, large loop excision of the transformation zone; ToC, test of cure.

^a^ Defined as HPV‐positive or atypical squamous cells of undetermined significance or worse.

^b^ Defined as HPV‐negative and normal cytology.

The 95% confidence intervals for relative proportions were calculated by assuming that their logarithms were approximately normally distributed.

Although we endeavored to report absolute numbers throughout the paper, these had to be suppressed following the national data analysis guidelines to avoid the risk of re‐identification in tabulations that included cells with <5 cases.

Statistical analyses were performed using SAS version 9.4.

## 3 RESULTS

### 3.1 Findings in the LLETZ specimen

A total of 1014 women underwent LLETZ at the age of 60–64 years during the study period, at a median age of 62 years (interquartile range: 61–63 years; Table 1). Among these, 72 women (7%) had a LLETZ specimen that was categorized as insufficient. The specimen showed at most CIN1 in 93 (9%) women, 567 (56%) specimens showed CIN2+, and 37 (4%) showed cervical cancer. The majority of cancer cases, 30/37 (81%), were squamous cell carcinoma (not tabulated). Among 660 women with CIN1+, 255 (39%) had free margins, 361 (55%) had involved or indeterminate margins, and 44 (7%) had unknown margin status.

### 3.2 Findings before the LLETZ procedure

In total, 869 (86%) women had a histological sample registered within 2 years prior to LLETZ (Table 2). These histology samples were insufficient for diagnosis, for unspecified reasons, in 109 (13%; 11% of all studied women) women. A pre‐LLETZ biopsy in a further 551 (63%; 54% of all studied women) women showed CIN2+, and in 13 (1%) it showed cervical cancer.

Among 567 women with CIN2+ in their LLETZ specimen, the worst histological finding in the two preceding years was also CIN2+ in 407 (72%) of cases (Table 3). Of the 37 women with a LLETZ specimen showing cervical cancer, 28 had a histological sample with CIN2+ registered in the previous 24 months. Among these 28 women, cervical cancer was detected in only seven cases.

### 3.3 Findings after the LLETZ procedure

At the first test‐of‐cure follow‐up test (median: 194 days, interquartile range: 169–226 days after the LLETZ procedure), 818 (81%) women had complete test‐of‐cure tests and were thus examined with both cytology and HPV testing (Table 4). In total, 406 (50% of those with complete test‐of‐cure tests, 40% of the studied population) women had a positive test‐of‐cure test, which could be either an HPV‐positive test or abnormal cytology, or both.

Post‐LLETZ histology was assessed within 24 months in 408 (40%) women (Table 4). In these women, the histological specimen was insufficient in 73 (18%) cases. A further 134 (33%, 13% of all studied women) had CIN2+, which included 19 cases of residual and 11 new cases of cervical cancer.

Among 567 women with CIN2+ in their LLETZ specimen, 248 (44%) had a new histological specimen in the ensuing 2 years (Table 3). Among these women, 103 (42%; 18% of all women with CIN2+ in their LLETZ specimen) showed persistence of CIN2+. Within 24 months after LLETZ, 108 (11%) women underwent a hysterectomy, among whom 60 (56%) had CIN2+ and 22 had cancer (20%). An additional 93 women had another LLETZ as the most invasive procedure performed (not tabulated), among whom 35 (38%) had CIN2+.

### 3.4 Subgroup of women with a positive HPV test and no other abnormalities prior to LLETZ

There were 108 (11%) women in the study with a LLETZ procedure that was only preceded by a positive HPV test and for whom no other abnormalities were recorded in the Patobank. This is a subset of the 145 women with no pre‐LLETZ histology reported in Table 2. Among these 108 women, 18 (17%) had CIN2+ in the LLETZ specimen, and 12 (11%) had CIN3+ including cancer (Table 5). After the LLETZ procedure, 95 (88%) had test‐of‐cure HPV tests performed, among whom around half, 53 (49%), continued to show positive results.

## 4 DISCUSSION

In Danish women in their sixties, many of those who underwent a LLETZ procedure had no prior clinically relevant abnormalities. Around four out of every five cancers detected in the LLETZ specimen were missed by the preceding biopsies. Despite LLETZ, a high proportion of these women had persistent abnormalities on HPV tests, cytology, and histology, including histologically confirmed cancer. Half of those presenting for test‐of‐cure testing had persistently positive HPV tests or abnormal cytology, and one in eight showed residual CIN2+. One in five women underwent another surgical intervention, half of which was a hysterectomy (not tabulated).

The dilemma of underdiagnosis versus overtreatment is especially important in older women. The women will no longer be monitored when they exit the screening program, and comorbidities, quality of life, and life expectancy become increasingly relevant. The indication for LLETZ in the case of CIN2+ is widely recognized^12^ which was also the case for 54% of LLETZ in our real‐world, population‐based study (Table 2). However, another 21% of women proceeded to LLETZ without a histological confirmation of CIN2+, in 11% the colposcopist unsuccessfully tried to obtain a valid histological diagnosis, and 14% of women had LLETZ without any recent histology. Although so many of these LLETZ were undertaken without prior evidence of high‐grade neoplasia verified on a biopsy, LLETZ specimens for the whole studied group showed CIN3+ in 35% of the women and cancer in 4% (Table 1). Histologically confirmed CIN2+ and CIN3+ were detected even in women whose only indication for a LLETZ procedure appeared to be a positive HPV test (Table 5). However, some of these LLETZ might have been performed due to visible disease, which we were unable to verify based on the Patobank data alone. Furthermore, only about one in five cancers present in the LLETZ specimen was also detected in pre‐LLETZ biopsies (Table 3). These data, therefore, indicate a significant and persistent reservoir of HPV infections and serious cervical lesions among a small proportion of women who are nearing the end of their screening eligibility, which in Denmark and several other countries ceases around age 65 years. The data also suggest that while (diagnostic) LLETZ will not always provide a definite answer, a reliable diagnosis might not be established without it.

A third of LLETZ specimens included in our study showed no sign of high‐grade neoplasia (Table 1). This proportion was high compared to younger Danish women, where only 5% of those with CIN2+ as an indication for LLETZ had no neoplasia in their LLETZ specimens.^13^ An explanation could be that colposcopy, as well as biopsies, are often insufficient due to the retraction of the transformation zone with increasing age^14^ and LLETZ is therefore used for diagnostic purposes. Another explanation for the seemingly liberal use of LLETZ could be that patients opt for LLETZ provided that it could reduce the risk of disease and require less follow‐up.^9^ However, the liberal use of LLETZ is associated with an increased risk of cervical stenosis in older women.^15^ The risk of cervical stenosis ranged from 1% to 15% across studies^15^, ^16^ Stenosis makes it more difficult to perform colposcopy, cytology, and histology, and insufficient samples will sometimes lead to a hysterectomy, which carries a risk of complications.^17^ Other common complications of cervical treatment include bleeding.^18^ Rare, but serious complications such as bladder injury^19^ have also been described. Overall, a study of >500 treatments found that almost 10% were associated with a complication.^20^

Notably, we found that around half of the women had positive test‐of‐cure tests after their LLETZ procedure (Table 4). This is high compared to the findings of a large systematic review, in which 28% of women aged ≥35 years remained HPV‐positive 6 months after LLETZ but with a large variation between the studies.^21^ Although this may suggest that LLETZ is more effective at removing HPV infections at a younger age, studies have provided inconsistent results.^22^, ^23^ In all, these data underscore the fact that older HPV‐positive women are a challenging group to manage and that LLETZ cannot be used to treat an HPV infection. This is important for healthcare providers who must make decisions about the continued management of women who are HPV‐positive but free from cytological or histological abnormalities. It is equally important for patients to understand that becoming HPV‐free may not always be a realistic expectation after LLETZ. Future research should focus on the long‐term risk of cervical cancer, particularly in older HPV‐positive women without histological abnormalities. Risk factors for persistent post‐treatment HPV infections should also be explored. If hysterectomy is the chosen treatment modality, the risks of vaginal HPV positivity should also be communicated to women.

The study included an unselected population from an entire country and described real‐world outcomes following test‐of‐cure management that were retrieved from a highly complete pathology register. Certain limitations, however, ought to be addressed. Although all women with cervical LLETZ were included, the study group was of limited size when stratified by clinically relevant characteristics. Furthermore, for the purpose of this study, we had no access to the information on women's sociodemographic characteristics other than their age or to other clinical data, including colposcopy findings, which could help explain the observed patterns. The frequency and severity of the abnormal findings may also depend on the reason for taking the initial samples: they are likely lower when women present for screening compared with women who present for persistent abnormalities or symptoms. Unfortunately, the reason for taking a sample is not registered reliably in the Patobank.

## 5 CONCLUSION

Our study suggested that (diagnostic) LLETZ and the subsequent follow‐up in women aged 60–64 may identify a significant number of women with high‐grade CIN and cervical cancer, including in women for whom prior biopsies did not confirm such abnormalities. However, despite having had a LLETZ performed, many of these women continued to show persistent infections and abnormalities and required additional follow‐up and treatment. At the minimum, the increasingly challenging nature of cervical abnormalities despite treatment in older age should be discussed with the women prior to undertaking such procedures.

## AUTHOR CONTRIBUTIONS

Julie Laub Erdal: Data curation, formal analysis, methodology, software, validation, writing—original draft, review and editing. Reza Rafiolsadat Serizawa: Funding acquisition for the cost of data retrieval, writing—review and editing. Matejka Rebolj: Methodology, writing—review and editing. Jeppe Bennekou Schroll: Data curation, formal analysis, methodology, project administration, software, supervision, validation, writing—original draft, review and editing. All authors: Conceptualization and approval for submission.

## FUNDING INFORMATION

Matejka Rebolj: Cancer Research UK (reference: C8162/A27047).

## CONFLICT OF INTEREST STATEMENT

Reza Rafiolsadat Serizawa is a consultant for Merck. Matejka Rebolj: The former Public Health England and the UK National Screening Committee provided funding for the epidemiological evaluation of various cervical screening studies; current and former members of various expert groups providing advice to the English Cervical Screening Programme; attended meetings with HPV test manufacturers; fees for attendance at advisory board and other meetings organized by Hologic, shared with employer, including travel cost reimbursement if applicable. Julie Laub Erdal and Jeppe Bennekou Schroll report no conflict of interest.

## ETHICS STATEMENT

This register‐study is based on routinely collected data from the Danish Pathology Databank, with no need to access individual electronic patient records. No contact has been made to the included individuals, their relatives, or treating physicians. Ethical approval by the National Committee on Health Research Ethics is therefore not needed for this study. The project was reviewed by the Knowledge Centre on Data Protection Compliance on behalf of the Danish Capital Region (reference number: VD‐2019‐95) on February 15, 2019.
